# Glutamate as a therapeutic strategy to promote liver regeneration

**DOI:** 10.1002/ctm2.70421

**Published:** 2025-07-21

**Authors:** María del Mar Rigual, Nabil Djouder

**Affiliations:** ^1^ Growth Factors, Nutrients and Cancer Group, Molecular Oncology Programme, Centro Nacional de Investigaciones Oncológicas (CNIO) Madrid Spain

Rigual MdM, Djouder N. Glutamate as a therapeutic strategy to promote liver regeneration. *Clin Transl Med*. 2025;00:e70421. https://doi.org/10.1002/ctm2.70421

The intrinsic regenerative capacity of the liver is crucial for recovery following injury, surgical resection, or transplantation. However, in patients with chronic liver disease or extensive hepatectomy, this capacity is often compromised, limiting therapeutic outcomes. Recent findings uncover a novel, metabolically driven mechanism of liver regeneration centred on glutamate signalling. Following hepatic injury, downregulation of unconventional prefoldin RPB5 interactor 1 (URI1) in pericentral hepatocytes leads to reduced glutamine synthetase (GS) activity and elevated systemic glutamate levels. This circulating glutamate activates bone marrow‐derived macrophages, which migrate to the liver, stabilise hypoxia‐inducible factor 1‐alpha (HIF1α), and secrete Wnt3, initiating YAP1‐driven hepatocyte proliferation and effective tissue repair. Importantly, glutamate supplementation was shown to robustly promote liver regeneration and increase survival in preclinical models, including cirrhosis and 90% hepatectomy. These findings identify a clinically actionable metabolic‐immune axis and suggest that oral physiological concentration of glutamate supplementation could serve as a safe, affordable adjunct therapy for patients undergoing liver resection, transplantation, or managing chronic liver failure. In addition, the potential development of URI1 inhibitors could offer an additional pharmacological approach to modulate this regenerative pathway. Together, this work represents a paradigm shift in regenerative hepatology, establishing glutamate as a key therapeutic driver of liver repair and opening the door to clinical trials aiming to validate its efficacy and safety in human patients.

## 1 WHY LIVER REGENERATION IS REQUIRED?

The liver is a vital organ responsible for metabolic homeostasis and detoxification, rendering it particularly susceptible to injury from alcohol, drugs, environmental toxins, and dietary factors. Remarkably, despite continuous exposure to harmful agents, the liver possesses a unique and robust regenerative capacity. Hepatocytes, the principal functional cells of the liver and the cells that shoulder the regenerative process, are typically quiescent under normal conditions but can rapidly proliferate in response to tissue damage.^1^

This regenerative process is crucial for recovery from acute damage and for maintaining liver mass and function.^2^, ^3^ However, after chronic or repeated injury, the liver regenerative capacity declines significantly, impairing the classic regenerative program and limiting the ability of hepatocytes to proliferate. This reduction in regenerative potential is a major clinical problem, especially for patients with chronic liver diseases or liver cancer requiring surgical resection, such as tumour removal or liver transplantation, where robust regeneration is essential for survival and long‐term function.^4^

Despite the well‐known capacity of the liver to regenerate, precise molecular mechanisms, factors, and particular hepatocyte populations that govern regeneration efficiency have remained incompletely understood.^4^ Traditional models have emphasised growth factors and cytokines, but recent research highlights the importance of metabolic and immune crosstalk.^1^ The study by Rigual et al., published in *Nature*, uncovers a rapid, glutamate‐mediated signalling axis between hepatocytes and immune cells, fundamentally reshaping our understanding of hepatic repair and opening new avenues for therapeutic intervention.^5^

## 2 GLUTAMATE AS A SIGNALING MOLECULE

**FIGURE 1:**
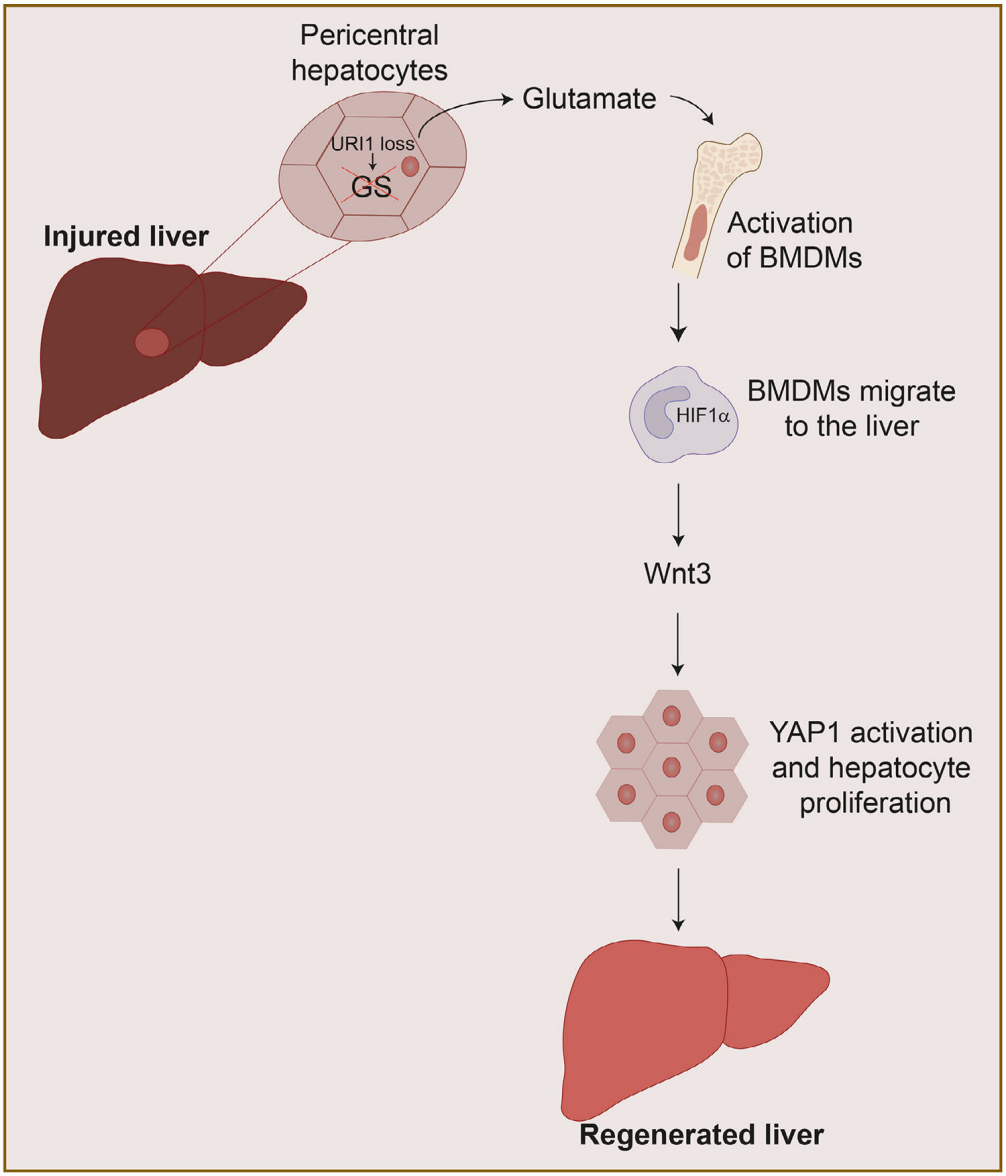
Schematic illustration of the glutamate‐mediated liver regeneration pathway. Following liver injury, loss of unconventional prefoldin RPB5 interactor 1 (URI1) in pericentral hepatocytes leads to decreased glutamine synthetase (GS) activity and increased glutamate release. Circulating glutamate activates bone marrow‐derived monocytes (BMDMs), which then migrate to the liver. In the hepatic environment, BMDMs stabilise hypoxia‐inducible factor 1‐alpha (HIF1α) and secrete Wnt3, promoting YAP1 activation and hepatocyte proliferation to restore liver mass.

This research uncovers a metabolic‐immune axis centred on URI1,^6^ a protein uniquely expressed in pericentral hepatocytes, localised around the central vein, also known as zone 3 hepatocytes. URI1 binds to and activates GS activity, a well‐known marker of pericentral hepatocytes surrounding the central vein, in turn decreasing glutamate and increasing glutamine in the bloodstream. Upon hepatic injury, URI1 expression is downregulated, GS activity is reduced, and circulating glutamate rises, which activates liver regeneration in animal models of acute and chronic liver injury^7^, ^8^ (Figure 1). Importantly, URI1‐overexpression dampens regeneration, while glutamate supplementation or GS knockout restores hepatic recovery in mice after standard partial hepatectomy (which involves removing 70% of the mouse liver). Notably, glutamate supplementation not only improved outcomes after partial hepatectomy but also proved beneficial in more severe models, such as cirrhosis and massive (90%) hepatectomy, underscoring the robustness and translational potential of this pathway.^5^

## 3 HIF1‐α MACROPHAGES KEY MODULATORS OF LIVER REGENERATION

Following liver injury, glutamate is released into the circulation and travels to the bone marrow, where it metabolically reprograms bone marrow‐derived monocytes, leading to stabilisation of HIF1α. These activated monocytes then migrate back to the injured liver, where HIF1α stabilisation induces the expression of Wnt3. Secreted Wnt3 subsequently activates the YAP1 signalling pathway in hepatocytes, promoting their proliferation and enabling efficient liver regeneration. This regenerative axis is initiated within minutes of hepatic injury, highlighting a rapid and tightly coordinated crosstalk between the liver and the immune system^5^ (Figure 1).

## 4 CLINICAL IMPLICATIONS AND FUTURE DIRECTIONS

The clinical implications of these findings are substantial. First, identifying glutamate as a key mediator of liver regeneration introduces a drug‐accessible axis for therapeutic activation of hepatic repair. Nutritional glutamate supplementation may emerge as a simple, cost‐effective adjunct to standard care for patients recovering from hepatectomy, awaiting transplantation, or suffering from chronic liver diseases. The next critical step is to translate these findings to humans and then design and conduct clinical trials to evaluate the safety, optimal dosing, and efficacy of glutamate supplementation in these patient populations. Additionally, exploring glutamate supplementation in combination with established regenerative or immunomodulatory therapies may yield synergistic effects and further improve patient outcomes.

The glutamate‐HIF1α‐Wnt3 axis may have broader relevance in regenerative medicine, potentially extending beyond the liver to other organs, including traditionally non‐regenerative tissues such as the pancreas, heart, or central nervous system. This axis represents a paradigm shift, emphasising the therapeutic potential of metabolic‐immune signalling in tissue repair.

Moreover, given that loss of URI1 has also been implicated in promoting intestinal regeneration,^9^, ^10^ ongoing research is actively focused on developing specific URI1 inhibitors as pharmacological tools to selectively and temporally activate this regenerative program when needed. Such targeted interventions could offer a novel and versatile therapeutic strategy not only for enhancing liver repair after injury or resection, but also for promoting regeneration in a wide range of acute and chronic degenerative conditions across multiple organs. Ultimately, harnessing this pathway may transform current clinical approaches to organ failure, transplantation, and tissue regeneration. In summary, the work of Rigual et al. provides a paradigm shift in our understanding of liver regeneration, highlighting the central role of metabolic‐immune interactions and offering promising new therapeutic strategies for enhancing hepatic repair in clinical practice.

## CONFLICT OF INTEREST STATEMENT

The authors declare no conflict of interest.
